# Soy-Based High-Protein Spheric Foods with the Appearance of Familiar Sugary Snacks

**DOI:** 10.3390/foods13081176

**Published:** 2024-04-12

**Authors:** Hiroyuki Yano, Rika Tanaka, Wei Fu

**Affiliations:** Institute of Food Research, National Agriculture and Food Research Organization, Ibaraki 305-8642, Japanfui528@affrc.go.jp (W.F.)

**Keywords:** 3D laser surface scanning, crispiness, meltability in the mouth

## Abstract

Excessive consumption of sugary foods increases the likelihood of obesity, as well as the preventable risk of lifestyle illnesses such as diabetes and cardiovascular diseases. Frequent intake of sweet snacks is considered to increase the risk of overweight/obesity in industrial nations. However, we cannot stop snacking against our better judgment. Therefore, in this study, we sought to develop high-protein, low-carb “mock snacks” to satisfy snack lovers’ appetites and nutrition. Soy protein-based, ball-shaped food products with 57.7% (*w*/*w*) protein and 3.6% sugar have been developed. The addition of canola oil made them melty in the mouth without sacrificing their crispiness. Moreover, evaluation of the surface topography of the “soy balls” by 3D laser scanning demonstrated their high degree of sphericity. Conclusively, the snacks developed here may be one of the healthy alternatives for the current sugary ones.

## 1. Introduction

Overconsumption of sugary foods triggers severe chronic diseases such as type-2 diabetes, obesity, and cardiovascular diseases [1,2]. The global population of diabetes amounts to about 463 million and is estimated to exceed 500 million by the year 2030 [3]. The prevalence of diabetes boosts health care costs, particularly spending per person on inpatient medical stays and prescribed pharmaceuticals [4]. Indeed, the inflation-adjusted direct medical expenditures for diabetes are estimated to have risen 7% from 2017 and 35% from 2012, respectively. The immense economic loss due to diabetes weighs on society through direct medical as well as indirect costs [4]. Moreover, studies on aged rats demonstrated that brain functions are highly susceptible to high-caloric diets with a negative impact on cognition and emotions such as anxiety, learning, and memory, and in the hippocampus, neurogenesis and neuroinflammation [5]. As we consume sugar, we feel pleasure with the release of dopamine, which tempts us to consume more sugar, leading to an increase in receptors for dopamine in the brain [6]. This vicious circle makes us addicted to sugar [7]. Additionally, the developmental trajectory of the human prefrontal cortex, a critical brain region for high-level cognitive function, as well as behavioral and self-control, does not mature functionally until the early twenties. Furthermore, dopamine, a neurotransmitter particularly abundant during adolescence, tunes the brain to learn rapidly about rewards and regulates aspects of neuroplasticity. Thus, adolescence is considered a vulnerable period to reward-driven behaviors such as consuming high-sugar “junky foods” [8].

Then, how can we stop reaching for sugary snacks? Experimental and longitudinal data show that increased dietary snack consumption contributes to energy intake in the diet as well as higher body mass levels [9]. However, since in-between-meal snacking has been casually incorporated into our daily lives, it is not easy to escape from the habit [10]. For those with overeating behavior and binge eating disorder (BED), mere confrontation with food triggers a conditioned response, that is, a cue-induced food craving [11]. The appearance of food products significantly determines food preferences and food choices [12]. The shape of the food is a key parameter that defines its visual attributes and plays a critical role in its consumer acceptability [13,14,15]. Thus, if high-protein, low-carb foods with a familiar look of sugary snacks are available, they can satisfy snack lovers’ appetites without abusing their health. Crispy wafer balls coated with chocolate are one of the representative favorites for snack lovers [16]. In this research, we explored the development of healthy snacks with the appearance of familiar sugary balls.

## 2. Materials and Methods

### 2.1. Materials

Food-grade soy protein isolate (SPI) was procured from NICHIGA, Japan Garlic Co., Ltd. (Takasaki, Japan). The SPI flour contained 85.0 g/100 g protein, 3.0 g/100 g lipid, and 1.0 g/100 g carbohydrate. Canola oil was purchased from Nisshin OilliO (The Nisshin OilliO Group, Ltd., Tokyo, Japan). Mineral water, with 30 mg/L hardness, was from Suntory (Osaka, Japan). Oxidized glutathione was from Nacalai Tesque (Kyoto, Japan).

### 2.2. Making Soy Balls

Soy dough was prepared using a YFA-201 food processor (Yamaden, Tokyo, Japan). In summary, 30 g of SPI and 70 g of water were mixed by kneading paddles for 40 s in the container of the food processor. In some cases, when canola oil was incorporated into the formulation of the dough, a portion of water was replaced with the same amount (weight) of canola oil to maintain the concentration of SPI in the dough. Small portions of the dough, each 1.5 g, were shaped into balls manually and were placed on a flat ceramic baking plate. Subsequent baking of the small ball-shaped doughs was carried out at 120–250 °C for 25 min, using an RE-S205 electric oven (Sharp Co., Osaka, Japan) and following the supplier’s instructions. Five soy balls were made for the respective experimental conditions.

### 2.3. Measurement of the Sphericity of Soy Balls

**Figure 1:**
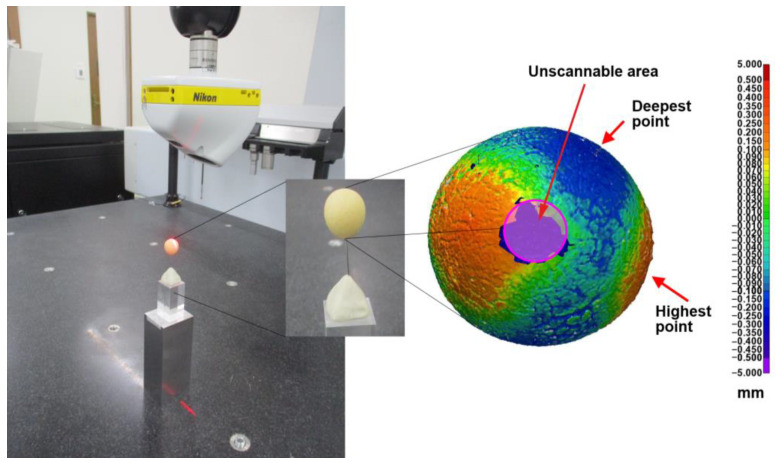
The 3D laser surface scanning setup of the soy balls (**left**) and an example of the scanned data (**right**).

The 3D laser surface scanning technique has been used in a broad range of industries: assessment of residual stresses in the laser powder bed fusion of metals [17], improvement of the rolling contact joint of biomechanical implants [18], or the creation of three-unit fixed partial dentures [19]. In this study, this technique was applied to evaluate the sphericity of the soy balls using a coordinate measuring machine, NIKON Altera 10.10.8 (LK Metrology Ltd., Derby, UK), equipped with a non-contact NIKON LC15Dx laser scanning probe (Left panel, Figure 1). Each soy ball, sustained in a vertical fashion by a thin wire, was subjected to measurement. Five soy balls were measured for the respective experimental conditions. The undulation (topology, sphericity) of each sample was quantitatively evaluated by post-processing and analysis of numerically extracted data using the dedicated software Polyworks Inspector 2020 IR8 (InnovMetric, Quebec, QC, Canada).

As the soy balls were not perfectly spherical, they had their respective “topology”, that is, high mountain and deep valley areas (Right panel, Figure 1). Therefore, first, the average diameter was calculated for each soy ball based on the scan data. Then, the standard deviation of the height was determined, which was considered an index for the “sphericity” of each ball. Moreover, the volume of each ball was calculated from the scan data, which were used to determine the specific volume (mL/g) in combination with the weight data of each ball. Just to mention that the vicinity area of the ball–wire junction was unscannable, so it was excluded from calculations (Right panel, Figure 1).

### 2.4. Nutritional Composition Analyses

All nutrient composition analyses of the soy balls were carried out in the Japan Food Research Laboratory (JFRL, Tokyo, Japan), one of the world’s largest testing service providers, according to its standard methods. The crude protein content was determined using the Kjeldahl method with a conversion factor of 5.71. Moisture, vaporing method; fat, Soxhlet method; dietary fibers, enzyme-gravimetric HPLC method (AOAC 2001.03); ash, direct incineration method; sodium, atomic absorption spectrometry. Sodium contents (*w*/*w*) were multiplied by 2.54 to calculate the salt contents of the food (*w*/*w*). The amount of sugar % (*w*/*w*) was obtained by calculation, that is, subtracting the sum of the % (*w*/*w*) of water, protein, fat, ash, and dietary fiber from 100% (*w*/*w*). Energy (kcal/100 g) was calculated from the amount of protein (4 kcal/100 g), fat (9 kcal/100 g), sugar (4 kcal/100 g), and dietary fiber (2 kcal/100 g). The experiments were repeated independently three times.

### 2.5. Sensory Evaluation on Crunchiness and Melting in the Mouth

Sensory evaluation to assess the crispiness and meltability in the mouth of the soy balls was also conducted in the JFRL. It was performed as a commissioned analysis, according to the guideline number JIS Z 9080:2004 of the Japan Industrial Standards Committee [20]. The panel included twelve trained analytical staff members, 10 females and 2 males, who were all employees of JFRL, where this sensory evaluation was conducted. All panelists passed the prerequisite tests on olfactory perception using T&T olfactometer reagents (Daiichi Yakuhin Sangyo, Tokyo, Japan). They also passed a taste sensation test using an aqueous solution of 0.4% (*w*/*w*) sucrose, 0.02% (*w*/*w*) citric acid, 0.13% salt, 0.05% (*w*/*w*) sodium glutamate, and 0.03% (*w*/*w*) caffeine. The panelists comprised experts with sufficient experience in the sensory evaluation of a wide range of food products, although they did not specialize in snacks.

Twelve panelists munched soy balls A and B and measured the crispiness and meltability in the mouth of B with reference to A. Each panelist compared the meltability in the mouth of the soy balls A and B, and the intensity was determined using a 7-point evaluation scale, −3 to +3, where −3, much worse; −2, worse; −1, slightly worse; 0, not different; +1, slightly better; +2, better; +3, much better. In the evaluation of crispiness, the words “worse”/“better” were replaced with “lower”/”higher”. Six panelists evaluated soy ball B after the evaluation of soy ball A, where the scores for soy ball A were set at 0. The other 6 panelists evaluated A after B. In this case, scores for soy ball B were set at 0. To prevent bias, soy balls A and B were randomly labeled as 948 and 012, respectively. All panelists were informed of the content of the experiment beforehand, voluntarily took part in the evaluation, and agreed to the collection of the statistical data. During this study, the rights and privacy of all participants were protected. Sensory evaluation was performed in a dedicated sensory booth that was quiet, comfortable, and air-conditioned. Panelists were provided with a relaxing environment and water for rinsing their mouths before the evaluation of the next sample.

## 3. Results and Discussion

### 3.1. Production of Soy Protein-Based, Ball-Shaped Food

A soy dough made of 30 g of SPI, 10 g of canola oil, and 60 g of water was broken down into small 1.5 g balls. Subsequent baking of the balls on a flat ceramic baking plate at 220 °C for 25 min in an electric oven yielded round and swollen spheric products. Below, the effects of oven temperature as well as the weight of the dough on the properties of the baked products are briefly introduced. Five balls were made for the respective experimental conditions.

**Figure 2:**
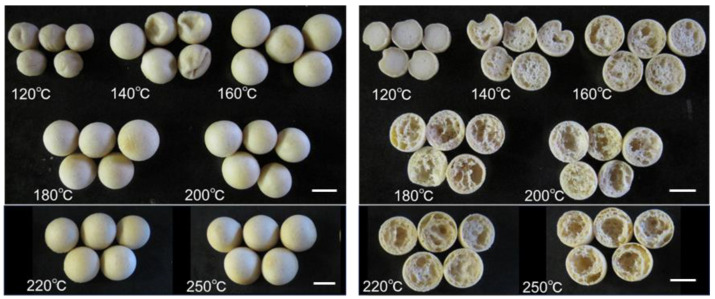
Effect of baking temperature on the external and internal appearances of soy balls. (**Left panel**), exterior appearance. (**Right panel**), cross-section. White bar, 10 mm.

When the baking temperature was lower than 120 °C, the dough (1.5 g) did not swell (Figure 2). At 140 °C, the dough swelled but shrank gradually when it was left at room temperature after baking. At 160 °C, the dough swelled into a ball shape, but the outer shell was soft, and the inside was moist. With a baking temperature greater than 180 °C, the outer shell was hard, and the inside was dry and had an air-celled interior structure.

As a preliminary study to investigate the texture of the soy balls, sensory evaluations on the crispiness and meltability in the mouth were investigated later in this paper to evaluate their applicability as food.

**Figure 3:**
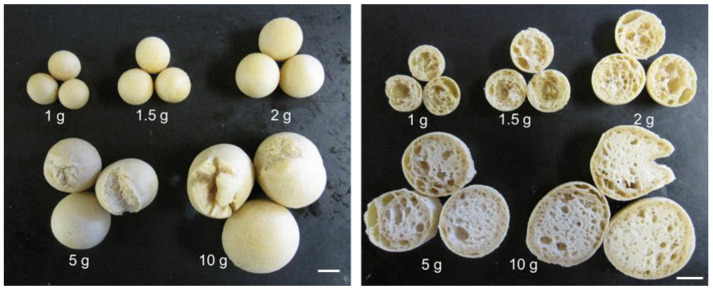
Effect of the dough weight on the external and internal appearances of soy balls after baking. (**Left panel**), exterior appearance. (**Right panel**), cross-section. White bar, 10 mm.

Next, the effect of dough weight on the properties of soy balls was explored (Figure 3). When the weight of the dough before baking was lower than 2 g, the outer shell of the baked dough became hard with an air-celled crispy interior. However, when the raw dough weighed more than 5 g, the envelope cracked during baking, and the inside was wet.

Based on these preliminary studies, the oven temperature and dough weight were set to 220 °C and 1.5 g, respectively.

### 3.2. Evaluation of Soy Ball’s Sphericity

Next, the effect of oil addition to soy dough on the shape of the soy balls was investigated. Table 1 summarizes the ingredient composition of soy doughs A, B, C, D, E, and F. Soy ball F contained oxidized glutathione (GSSG) to show the effect of the disulfide bonds of protein on the formation of the spherical structure of soy balls, which will be mentioned later in this paper. Five balls were made for the respective experimental conditions.

**Figure 4:**
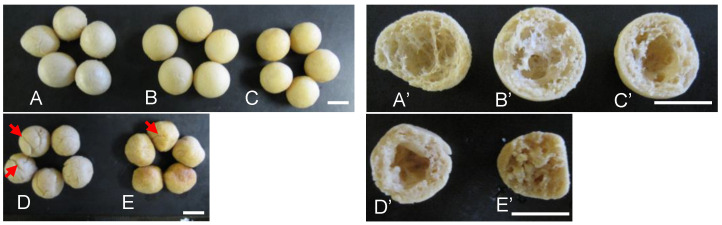
Addition effect of oil to soy dough on the appearance of soy balls. (**Left panel**), exterior appearance. (**Right panel**), cross-section. White bar, 10 mm. Red arrows, cracks. (**A**–**E**), exterior appearance of the respective soy balls whose ingredient composition is shown in Table 1; (**A’**–**E’**) cross-section of the respective soy balls (**A**–**E**).

Without oil, the soy balls were generally spheric with some “baroque pearl”-like examples (Figure 4A). However, when 10–20% (*w*/*w*) of canola oil was added to the dough (Figure 4B,C), the appearance became more spheric. With a 30–40% (*w*/*w*) addition of oil (Figure 4D,E), the shapes became distorted with cracked outer shells.

**Figure 5:**
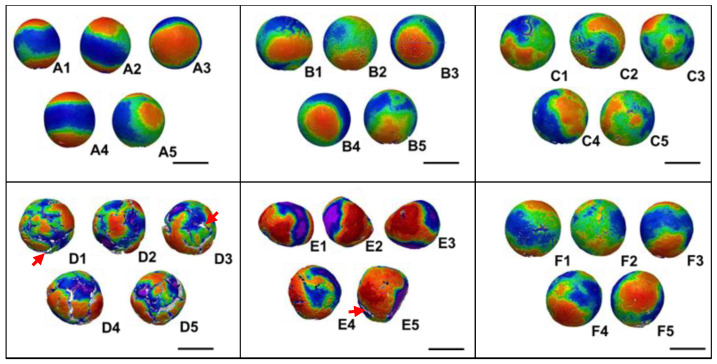
Laser scanning results of the soy balls. Bar, 10 mm. Red arrows, cracks. Scanning was performed for soy balls (**A**–**F**) which were produced independently for five times following the ingredient composition shown in Table 1. Thus, each scan data is labeled as A1–5, B1–5, C1–5, D1–5, E1–5 and F1–5. The color scale is shown in the Figure 1.

Then, the soy balls were subjected to laser scanning (Figure 5). Five balls #1–5 were subjected to the scan for the respective conditions A–F. As shown in Figure 1, red areas demonstrate high mountain regions, whereas blue areas show low valley areas. With the appearance and color distribution, it was speculated that B and C were the most spheric (Figure 5).

**Figure 6:**
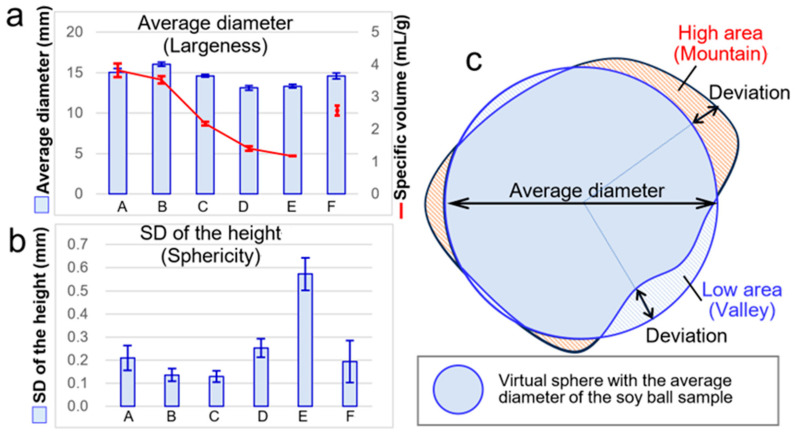
Shape measurement of the soy balls. (**a**), blue rectangles, average diameter; red line, specific volume; (**b**), standard deviation of the height; (**c**), explanatory drawing of the measurements. SD, standard deviation. (A–F), respective soy balls whose ingredient composition is shown in Table 1.

Next, we sought to assess the size of the soy balls (Figure 6). The blue vertical rectangles of Figure 6a show the average diameter (mm) of the respective soy balls calculated by the scan data. The longest diameter, 16.05 ± 0.23 mm, was obtained for B, whose dough before baking contained 10% (*w*/*w*) of canola oil. The red line plots show the specific volume (SV, cm^3^/g) of the respective soy balls. The SV decreased with the increased amount of added oil. The SV of A and B was more than 3.5 mL/g, equivalent to that of wheat bread. Thus, in this sense, we considered A and B as candidates for “light” snacks.

To compare the sphericity of the soy balls, the standard deviation (SD) of each ball’s height, from the respective soy ball’s average diameter, that is, the average height, was calculated from the scan data (Figure 6b). Soy balls B and C had the lowest SDs, 0.136 ± 0.028 mm for B and 0.130 ± 0.024 mm for C, that is, the highest sphericity (Figure 6c). Conclusively, soy ball B was the lightest and had the most spheric structure among the six soy balls. In this experiment, when canola oil was added to the formulation of a dough, the same amount of water was subtracted from the dough to keep the concentration of SPI in the dough. However, as the formulation of the dough can affect the texture and topography of the baked snacks, further test productions based on the systematic formulation of the dough should be explored to find the best formulation of the soy balls. We shaped the ball manually, because the doughs, before baking, had different physical properties such as stickiness and softness, and it was not easy to shape the doughs uniformly with a machine. Although we made efforts to shape the doughs into spheric structures, the manual shaping process might have affected the topography of the snack ball. A preliminary trial to make soy-based spheric foods was mostly accomplished in this Communication. However, further studies are needed and are in progress in our lab to facilitate more sophisticated production of soy balls.

### 3.3. Nutritional Evaluation of Soy Balls

Next, the nutritional composition of soy ball B was determined (Table 2). The experiments were repeated independently three times.

Soy ball B contained 57.7% protein and 3.63% sugar. The protein/sugar ratio was as high as 15.9. It also contained 25.07% (*w*/*w*) of lipid, which has been known to have a low glycemic index; thus, the effect on postprandial glucose and insulin responses is negligible [21,22]. Compared to an example of sugary balls containing 3% protein and 16% sugar [16], the soy ball developed here is high in protein and low in carbohydrates. Additionally, soybean-based food is reported to maintain satiety and suppress hunger compared to wheat-based food [23]. Moreover, in our unpublished studies, the replacement of canola oil with high-ω-3 perilla oil or linseed oil did not significantly change the exterior/interior structures of the soy balls. Thus, soy balls are considered the ideal health snacks to replace high-carbohydrate sugary ones. They also conform to the SDGs because soy protein is a reuse of oil cakes [24].

### 3.4. Sensory Evaluation of the Soy Balls’ Crunchiness and Meltability in the Mouth

**Figure 7:**
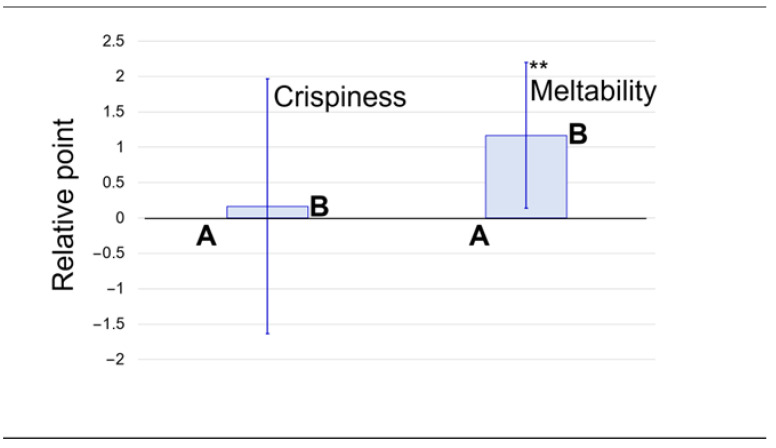
Comparison of the soy balls’ crispiness and meltability in the mouth by sensory evaluation. A and B are soy balls whose ingredient composition is shown in Table 1. **: significant difference (*p* < 0.01).

Next, the difference in texture was compared between soy balls A and B. Twelve trained panelists evaluated the crispiness and meltability in the mouth of B with reference to A. As shown in Figure 7, the addition of 10% canola oil to soy dough significantly increased the meltability of the baked product without sacrificing the crispiness. Crispiness is one of the critical factors in the enjoyment of many foods [25]. As aspiration, that is, accidental swallowing, is fatal for elderly people [26], high-protein low-carb snacks with both crispiness and high meltability are ideal foods for elderly people. However, the results are preliminary considering so many other important attributes. Although the soy balls are still in the experimental stage, their palatability as food will be improved by revising the formula, processing conditions, and flavoring.

### 3.5. Presumption of the Swelling Mechanism

**Figure 8:**
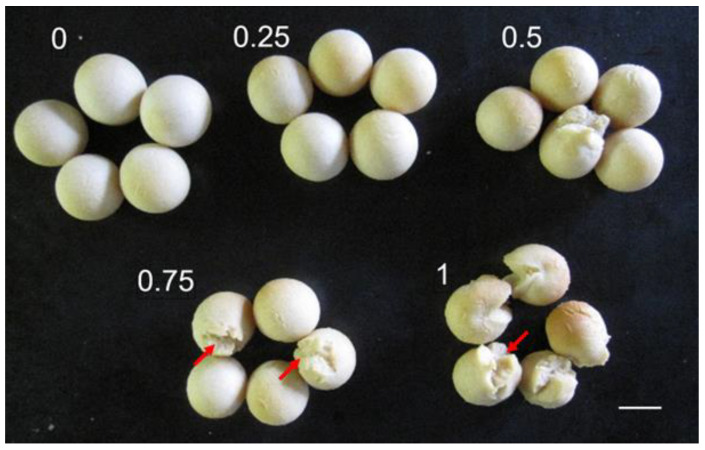
Effect of oxidized glutathione (GSSG) addition to soy dough on the exterior appearance of soy balls. Numbers; relative amount of addition of GSSG (*w*/*w*, %). White bar, 10 mm. Red arrows, cracks.

We have reported so far that sulfhydryl compounds/enzymes such as glutathione, cysteine, and thioredoxins play critical roles in cereal biochemistry as well as food processing [27,28]. Recently, the combination of soy protein and egg white/hemp protein has been shown to make a membrane structure in which disulfide crosslinking among proteins plays critical roles [29,30]. So, as a preliminary study to see the effect of sulfhydryl compounds, GSSG was added to soy dough B, which was subjected to baking at 220 °C for 25 min. Five soy balls were made for the respective experimental conditions. As shown in Figure 8, the addition of GSSG (0.75%, *w*/*w*) made the outer shell tend to rip. Also, the sphericity as well as the diameter decreased (Figure 6a,b). Thus, it is highly possible that disulfide crosslinking among soy proteins plays a critical role in the construction of the spheric shell structure. Further mechanism studies are in progress in our lab.

## 4. Conclusions

High-protein, low-carb snacks were developed with soy protein as a major ingredient. When 1.5 g amounts of dough composed of 30% (*w*/*w*) SPI, 10% (*w*/*w*) canola oil, and 60% (*w*/*w*) water were molded manually into balls and baked in an electric oven at 220 °C for 25 min, the spheric products contained 57.7% (*w*/*w*) protein and 3.6% (*w*/*w*) sugar. The diameter of the soy balls was about 16 mm, and the SD of the diameter was as low as 0.136 ± 0.028 mm. The specific volume, an index for the lightness of food, was as high as 3.5 mL/g, comparable to general wheat bread. Sensory evaluation of the soy balls by trained panelists demonstrated their high meltability in the mouth without sacrificing their crispiness. The snacks, with the appearance of familiar starch-based snacks such as crispy wafer balls, may be an alternative to the conventional sugary ones to satisfy snack lovers’ appetites and nutrition.

The preliminary experiments performed in this Communication paved the way for the development of high-protein, low-carb spheric snacks. However, a greater variety of experimental conditions should be explored for practical applications because the formulation of snacks affects their texture as well as topology. Thus, issues to be addressed in future investigations include comprehensive formulation studies to optimize the manufacturing process of soy balls. Moreover, instrumental measurements of the physical properties of soy balls are indispensable along with further sensory evaluations. These challenges will contribute to the improvement of the quality of the soy balls to realize the industrialization of new healthy snacks welcomed by a wide spectrum of consumers.

## 5. Patents

Part of this work has been used for a patent application in Japan No. 2023-174471, filed on 6 October 2023.

## Figures and Tables

**Table 1:** Ingredient composition of each soy ball.

	A	B	C	D	E	F
SPI (g)	30	30	30	30	30	30
Canola oil (g)	0	10	20	30	40	10
Water (g)	70	60	50	40	30	59.25
GSSG (g)	-	-	-	-	-	0.75

**Table 2:** Nutritional composition of soy ball B.

Nutrition	Average Amount (N = 3)
Moisture (g/100 g)	9.73 ± 0.40
Protein (g/100 g)	57.70 ± 0.0
Fat (g/100 g)	25.07 ± 0.15
Ash (g/100 g)	3.07 ± 0.06
Carbohydrate (g/100 g)	4.43 ± 0.31
***Sugar* (g/100 g)**	***3.63* ± *0.15***
***Dietary fiber* (g/100 g)**	***0.80* ± *0.17***
Energy (kcal/100 g)	472.55 ± 2.08
Salt (g/100 g)	1.58 ± 0.01

The amount of sugar and dietary fiber indicated in bold Italics are both breakdown of the carbohydrate amount.

## Data Availability

Data are contained within the article.
